# Next chapter for the Annals of Noninvasive Electrocardiology

**DOI:** 10.1111/anec.13019

**Published:** 2022-11-18

**Authors:** Wojciech Zareba

**Affiliations:** ^1^ University of Rochester Medical Center Rochester New York USA

For the last 11 years, I had a profound pleasure to serve as Editor‐in Chief, and my wife, Grazyna Zareba, PhD, assisted this activity as Managing Editor of the Annals. We have worked for the Annals with a purpose to serve and support the scientific and educational mission of the International Society for Holter and Noninvasive Electrocardiology (ISHNE). I would like to start by thanking my wife for her tireless work on the journal that made my life so much easier and we very much enjoyed working together, capitalizing on our prior collaborative experience with Cardiology Journal, which we help launch in 2006 and managed for several years.

Over the last decade, electrocardiology significantly evolved with increasing interest in new technologies acquiring ECG signals using patches, watches, stethoscopes, and other unconventional ECG devices. More than 120 years after the Einthoven's initial ECG recordings, the field of electrocardiology is thriving as the most frequently used test in cardiology if not entire medicine. Nowadays, progress in ECG acquisition devices is paralleled by advances in ECG signal processing becoming more sophisticated, further supported by machine learning and other computerized solutions. ECG does not know the borders or limits, and this is why our journal has been receiving manuscripts from all‐over‐the‐world on a multitude of interesting topics.

We were privileged to work with the John Wiley & Sons, Inc. (known as Wiley), the global publisher (founded in 1807) producing our journal with publisher's offices in Beijing and with production offices in New Delhi, with which we successfully collaborated for over decade. Daily tasks related to the Annals completed by my wife in the evening in Rochester, NY, were picked up by our collaborators in New Delhi or Beijing in their morning hours to reach the common goal: to make sure that every issue of the journal is up to the highest standards. We are very grateful to many colleagues from Wiley for their devoted work on the Annals.

**FIGURE 1:**
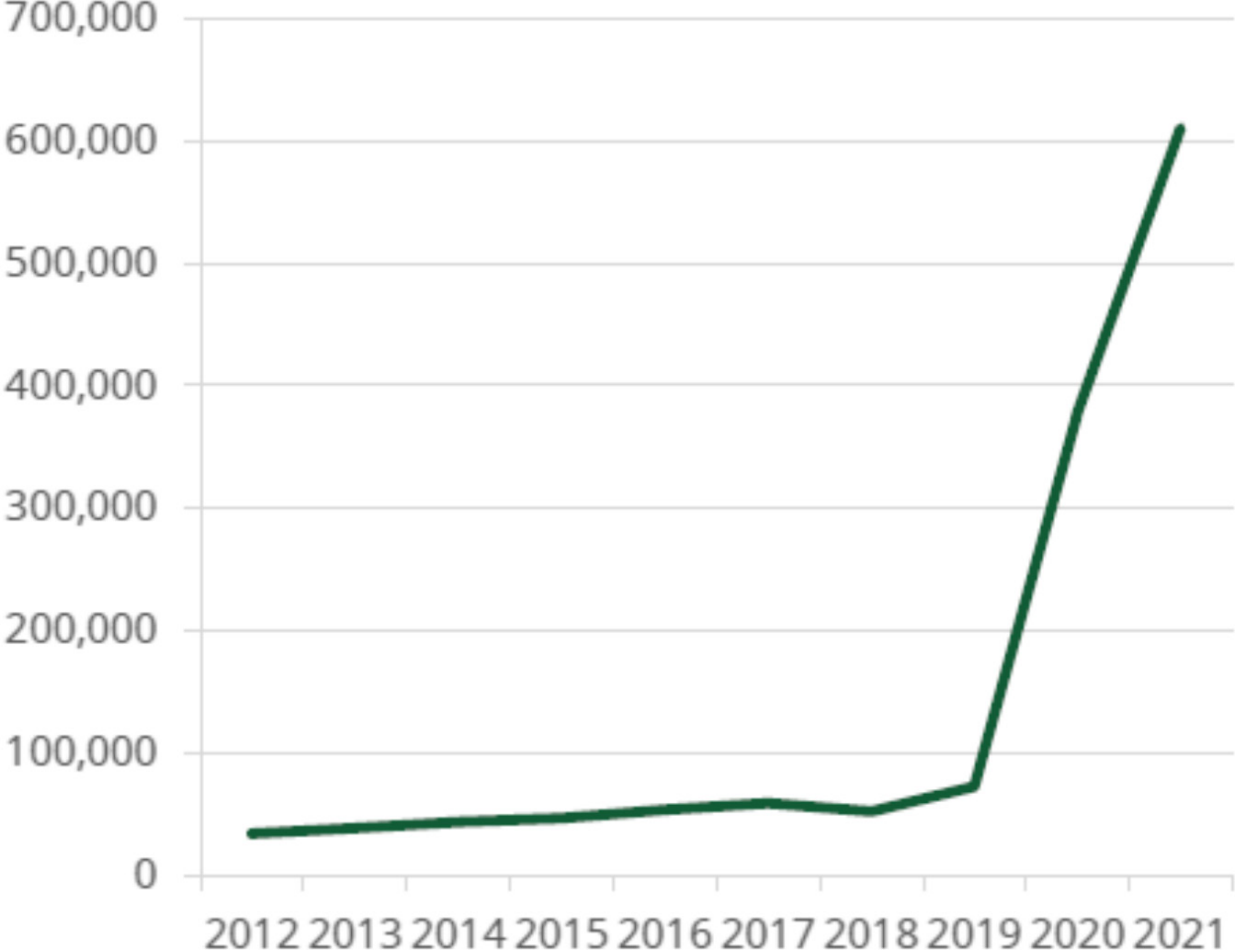
Chart showing the increase in the number of full‐text article downloads for the Annals in the period 2012–2021. The total includes usage on Wiley Online Library, PubMed, and other third party databases. Downloads via Wiley Online Library increased by 35.4% in 2021; this compares with an increase by 21.5% across all Wiley journals in the cardiovascular disease subject area. *Source*: Wiley Publisher's Report for the Annals of Noninvasive Electrocardiology.

**FIGURE 2:**
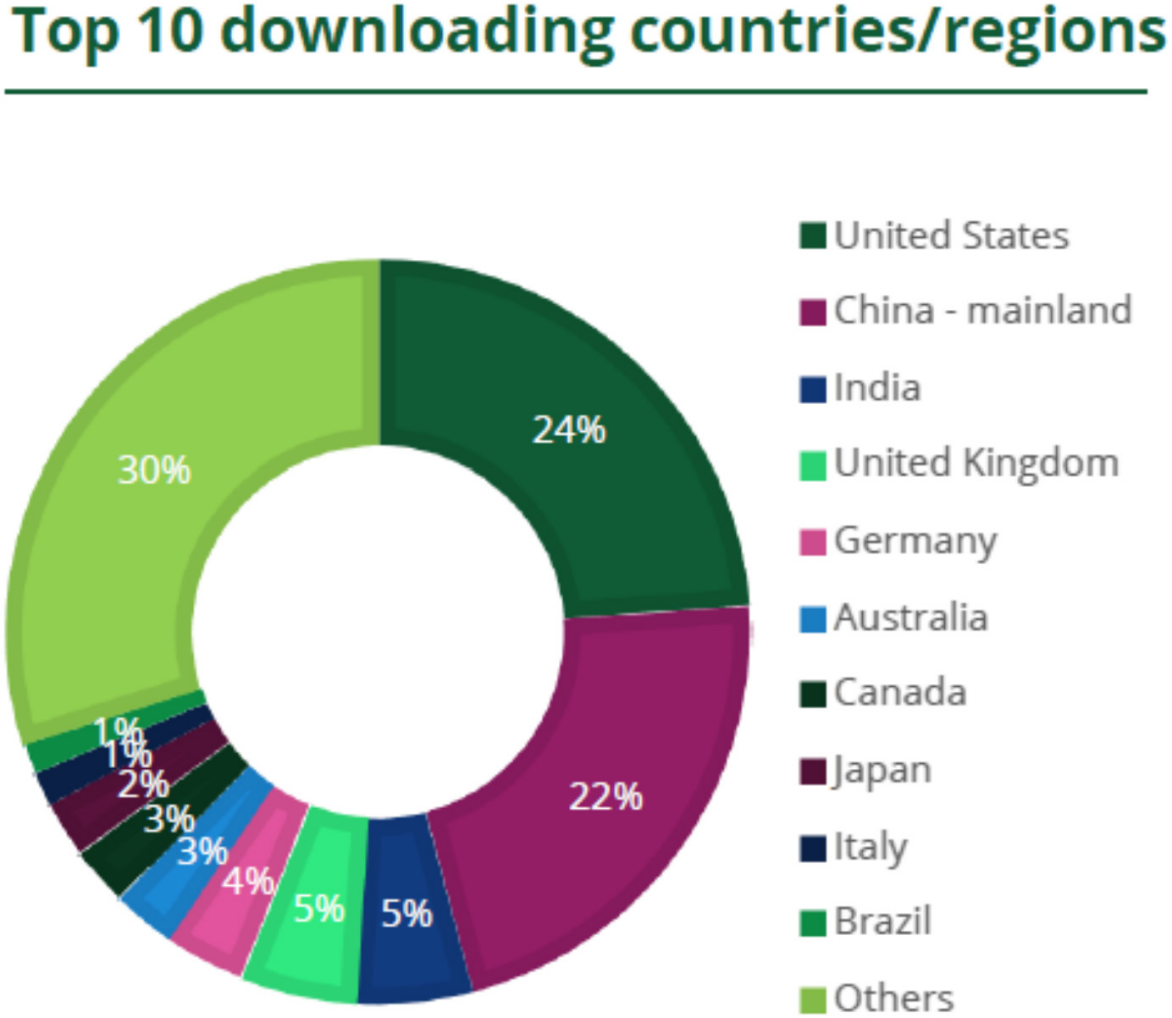
Chart showing the top 10 countries/regions from which articles in the Annals were downloaded via Wiley Online Library in 2021 and the percentage each country/region contributed to total usage. All other countries/regions are combined under “others.” *Source*: Wiley Publisher's Report for the Annals of Noninvasive Electrocardiology.

Our journal is gaining popularity especially since the Annals became in 2020 an open access journal. The annual 2021 Publisher Report (released by Wiley in June 2022) demonstrates a very significant increase in the full‐text article downloads reaching over 600,000 in 2021 (Figure 1) and with worldwide dissemination of science and education (Figure 2). This popularity should be credited to the authors submitting their papers to our journal, to the Editorial Board and to numerous devoted reviewers of the manuscripts, to whom we are very thankful.

But, there is a need for change since progress cannot be made in any field without change, and the change for the better! The next chapter for the Annals will be written by Dr. Mark Haigney, new Editor‐in‐Chief of the Annals, who has served over the years as Associate Editor of the journal. Dr Haigney, whom I have a great pleasure to know for 25 years, is not only a dear friend and colleague, but he is an eminent scholar and the expert in the field of electrocardiology, electrophysiology, cardiology, pharmacology, and several other fields of interest. Dr. Haigney is Director of Cardiology at the Uniformed Services University of the Health Sciences, Bethesda, MD, where he also leads Military Cardiovascular Outcomes Research (MiCOR) program. We are all pleased with Dr. Haigney, agreeing to serve as the next Editor‐in‐Chief of the Annals, the journal entering its 28th year of publication, which will be thriving for many years to come under Mark's leadership.

